# In-utero HIV exposure and postnatal growth to 8 years of age: a prospective cohort study in the Western Cape, South Africa

**DOI:** 10.1016/S2352-4642(26)00121-5

**Published:** 2026-09

**Authors:** Angela M Bengtson, Jennifer A Pellowski, Maresa Botha, Tiffany Burd, Lesley Workman, Elizabeth Goddard, Dan J Stein, David Burgner, Toby Mansell, Heather J Zar

**Affiliations:** a Department of Epidemiology, Emory University School of Public Health, Atlanta, GA, USA; b Department of Research Administration, Hartford Hospital, Hartford, CT, USA; c Department of Paediatrics and Child Health, Red Cross War Memorial Children's Hospital, Cape Town, South Africa; d South Africa Medical Research Council Unit on Child and Adolescent Health, University of Cape Town, Cape Town, South Africa; e South Africa Medical Research Council Unit on Risk and Resilience in Mental Disorders, University of Cape Town, Cape Town, South Africa; f Department of Psychiatry and Mental Health, Neuroscience Institute, University of Cape Town, Cape Town, South Africa; g Murdoch Children's Research Institute, Royal Children's Hospital, Parkville, VIC, Australia; h Department of Paediatrics, University of Melbourne, Parkville, VIC, Australia

## Abstract

**Background:**

The global scale-up of antiretroviral therapy for people living with HIV has led to an increase in children who are HIV-exposed uninfected (HEU), almost a quarter of whom lived in South Africa in 2024. These children might have suboptimal growth compared with children who are HIV-unexposed (HU), but few data are available beyond early childhood. We therefore evaluated differences in anthropometry by HIV exposure status from birth or from age 6 weeks, up to 8 years.

**Methods:**

The Drakenstein Child Health Study—a prospective, longitudinal cohort study in the Western Cape of South Africa—recruited adult (age ≥18 years) women with and without HIV at 20–28 weeks’ gestation from two primary health-care centres during routine antenatal care. Women and their liveborn children were followed up at least annually until age 8 years. The primary outcome was differences in anthropometry at birth or age 6 weeks and postnatally by HIV exposure, which was assessed at least annually by trained study staff and converted to sex-specific weight-for-age Z scores (WAZ), length-for-age or height-for-age Z scores (HAZ), and BMI-for-age Z scores (BMIZ) per WHO growth standards. All outcomes were assessed in all children with anthropometry data after birth. Stunting (HAZ <–2 SD from age 12 months) and overweight (BMIZ >2 SD from age 6 months) were secondary outcomes. Mixed-effects models adjusted for size at birth and prenatal covariates were used to estimate overall and age-specific associations between in-utero HIV-exposure status and WAZ, HAZ, BMIZ, stunting, and overweight from age 6 weeks to 8 years.

**Findings:**

1225 pregnant women were enrolled from March 5, 2012, to March 31, 2015. 88 women were ineligible or withdrew and the remaining 1137 delivered 1143 livebirths, of whom 71 children were ineligible or withdrew. The remaining 1072 children (HEU 236 [22·0%] *vs* HU 836 [78·0%]) were enrolled and followed up until age 8 years. Mean birthweight was 3035 g (SD 592; HEU 3012 g [598] *vs* HU 3041 g [590]) and 168 (15·7%) of 1070 infants were preterm (HEU 43 [18·3%] of 235 *vs* HU 125 [15·0%] of 835). In overall multivariable estimates, children who were HEU had lower overall WAZ (marginal difference –0·16 [95% CI –0·32 to –0·01]) and HAZ (–0·26 [–0·41 to –0·11]) from age 6 weeks to 8 years compared with children who were HU. Age-specific differences were largest before age 6 months for WAZ and before age 3 years for HAZ. In overall multivariable estimates, there was no association between HIV exposure and BMIZ (–0·02 [–0·17 to 0·12]), stunting (0·05 [–0·04 to 0·13]), or overweight (–0·01 [–0·04 to 0·02]) from age 6 weeks to 8 years.

**Interpretation:**

Compared with children who were HU, children who were HEU had lower overall WAZ and HAZ from age 6 weeks to 8 years, with the largest differences in early childhood. Early-life growth deficits are predictors of adverse health outcomes in later life; therefore, future work should focus on understanding the causes of growth deficits to improve health outcomes for children who are HEU. Interventions to optimise growth for all children in South Africa remain crucial.

**Funding:**

The Eunice Kennedy Shriver National Institute of Child Health and Human Development, The Gates Foundation, and the National Health and Medical Research Council (Australia).

## Introduction

The global scale-up of antiretroviral therapy (ART) for people living with HIV has led to an increase in the number of children who are HIV-exposed but uninfected (HEU). Of the 15·7 million children HEU in 2024,^1^ nearly a quarter live in South Africa,^2^ which has the highest burden of HIV in the world. A 2025 meta-analysis found that, despite being born HIV-uninfected, children who are HEU might be more likely to have suboptimal growth than children who are HIV-unexposed (HU), even in the era of universal ART.^3^ Impaired growth in early life is associated with worse developmental, educational, economic, and cardiometabolic health outcomes in childhood and later life.^4^ Improving growth outcomes for children who are HEU has been identified as a key public health priority to ensure that they are not just living HIV-free, but are thriving.^5^ The meta-analysis noted there were few data comparing growth outcomes in children who are HEU or HU beyond age 2 years in the era of universal ART and prolonged breastfeeding^3^ to inform public health strategies to optimise growth.

**Research in context**

**Evidence before this study**

We searched PubMed for full-text articles in English published from Jan 1, 2015, to April 1, 2026, using the search string (“HIV exposure”) OR (“children born HIV free”) AND (“growth” or “anthropometry” or “birth”), to identify studies examining associations between in-utero HIV exposure and growth or anthropometry outcomes. We found 49 studies examining HIV exposure status and anthropometry, including two systematic reviews and meta-analyses from 2025 that focused on how in-utero HIV exposure affects postnatal growth or anthropometry. Compared with postnatal growth, fewer studies reported on differences in anthropometry at birth by HIV exposure status, with available evidence showing lower weight-for age Z scores (WAZ) and length-for-age Z scores (HAZ) at birth in children who are HIV-exposed but uninfected (HEU) compared with children who are HIV-unexposed (HU). Evidence varied about the associations between HIV exposure and reduced growth or anthropometry measures postnatally, with the most consistent evidence indicating that children who were HEU had lower postnatal length or height. Available studies predominantly focused on growth and anthropometry outcomes within the first 2 years of life. A 2025 meta-analysis found only two studies examining differences in anthropometry between ages 24 months and 60 months by HIV exposure status. Understanding how HIV exposure affects growth beyond age 2 years and into childhood is essential to optimise growth outcomes for children who are HEU.

**Added value of this study**

Among children participating in the Drakenstein Child Health Study (DCHS), children who were HEU and HU did not differ in birthweight, birthweight Z score, birth length Z score, or gestational age at birth. Postnatally, compared with children who were HU, children who were HEU had lower HAZ and WAZ from age 6 weeks to 8 years. Lower HAZ and WAZ did not translate into an increased risk of stunting or being overweight until age 8 years for children who were HEU compared with children who were HU. Both HEU and HU groups had mean WAZ and HAZ lower than mean WHO reference standards from age 6 weeks to 8 years. Our findings provide some of the first longitudinal evidence of growth deficits in HAZ and WAZ emerging after birth and persisting to age 8 years for children who are HEU.

**Implications of all the available evidence**

Despite starting from a similar size at birth in the DCHS cohort, children who were HEU had lower overall HAZ and WAZ from age 6 weeks to 8 years than those who were HU, with the largest differences observed in early childhood. Drivers of fetal and postnatal growth in children who are HEU might differ, with early childhood representing an important period for intervention to optimise growth and ensure children are living HIV free and thriving. WAZ and HAZ were often lower than mean WHO reference standards throughout for both HEU and HU groups, indicating that interventions to optimise growth for all children within this setting remain essential.

The mechanisms underlying suboptimal growth in children who are HEU are unclear but likely multifactorial, involving maternal, biological, behavioural, social, and structural factors. Infants who are HEU might be more likely to be born smaller,^6^, ^7^ breastfed for a shorter duration,^8^ and have a higher prevalence of infections in early life than children who are HU,^9^ all of which could affect growth. Among children who are HEU, maternal immunological status—including viral load suppression^10^ and timing and type of ART^11^, ^12^—might also influence growth. Growth patterns in children who are HEU might also differ by sex.^13^ Whether deficits in growth among children who are HEU in early life persist into later childhood and their long-term implications for health and development remain unclear.^5^, ^14^ To better understand how in-utero HIV exposure affects growth beyond early childhood, longitudinal data on children who are HEU or HU who are followed up from birth into childhood are needed.

To address this gap, we examined differences in anthropometry at birth and from age 6 weeks to 8 years by HIV exposure status in a birth cohort of children who were HU or HEU and had high ART exposure during gestation in South Africa. We also explored differences by breastfeeding status and sex and, among children who are HEU, we explored associations between maternal HIV and ART characteristics with anthropometry.

## Methods

### Study design and participants

In this prospective, longitudinal cohort study, data came from the Drakenstein Child Health Study (DCHS), a longitudinal birth cohort of children who are HEU or HU from two peri-urban communities in Paarl, South Africa.^15^ The study communities include a stable population (eg, little immigration or emigration) of approximately 200 000 people and are characterised by a high prevalence of poverty, substance use, and HIV infection.^15^ The study communities have a well established public health-care system that includes primary care; antenatal, obstetric, and HIV care; and prevention of vertical HIV transmission services available free of charge in accordance with national guidelines.^16^ The DCHS team includes community workers from the local communities, and several other members of the research team have lived experience of working and providing clinical care to South African children who are HEU or HU.

Details of the DCHS have been published previously.^15^ Pregnant women were recruited between March 5, 2012, and March 31, 2015, from two primary health-care clinics: Mbekweni (serving a predominantly Black African community) and TC Newman (serving a mixed-ancestry community). Mothers were enrolled in the DCHS while attending routine antenatal care and were prospectively followed up at least annually until their children reached age 8 years. Women were eligible for the study if they were 18 years or older, between 20 and 28 weeks’ gestation, planned attendance at one of the two recruitment clinics, and intended to remain in the area for at least 1 year.^15^, ^17^ Women with HIV enrolled into the DCHS received predominantly efavirenz-based ART during pregnancy in accordance with South African HIV guidelines at the time.^18^ Women and their children were followed up through delivery and at least annually thereafter until age 8 years.^15^ Study visits took place at primary health-care clinics but were separate from routine care. Additional cohort information is provided in appendix 1 (p 2).

The DCHS was approved by the Faculty of Health Sciences, Human Research Ethics Committee, University of Cape Town (401/2009) and by the Western Cape Provincial Health Research Committee. Mothers provided written informed consent at enrolment and were re-consented annually. Consent was done in the mother's preferred language: English, Afrikaans, or isiXhosa. Maternal consent (before age 7 years) and individual assent (from age 7–8 years) were obtained annually from children depending on their neurocognitive ability, which was longitudinally assessed using different scales.^19^

### Procedures and outcomes

The exposure of interest was in-utero HIV exposure status. Pregnant women with unknown HIV status at study enrolment underwent HIV testing as part of routine care.^16^ Those who tested negative for HIV at enrolment were re-tested in the third trimester when feasible and approximately every 3 months while breastfeeding as part of routine clinical care. Infants exposed to HIV were given nevirapine syrup prophylaxis for at least 6 weeks and tested for HIV at age 6 weeks, 6–12 months, 18 months, and if they appeared unwell under national guidelines as part of routine care.^16^

The primary outcome was differences in anthropometry at birth and postnatally by HIV exposure status over time and was assessed in all children with anthropometry data after birth. Anthropometry, including length (age <24 months) or height (age ≥24 months) and weight, was assessed at birth and at least annually at all study visits by trained study staff using standardised protocols and regularly calibrated equipment as per WHO guidelines.^20^ Continuous measures at birth were converted into sex-adjusted birthweight and birth length Z scores using WHO growth standards for term births (age ≥37 weeks)^21^ and the 2013 Fenton standards for preterm births (<37 weeks’ gestation).^22^ Postnatal anthropometry assessments were converted to age-adjusted and sex-adjusted weight-for-age Z scores (WAZ), length-for-age or height-for-age Z scores (HAZ), and BMI-for-age Z scores (BMIZ) based on WHO growth standards for term births (appendix 1 pp 2–3).^21^ WAZ and HAZ measures were adjusted for infants born preterm through to age 50 weeks using the 2013 Fenton standards.^22^ BMIZ are not defined for infants born preterm and data from those born preterm were therefore excluded before age 50 weeks.^22^ The secondary outcomes were assessed in all children who had anthropometry data after birth, for which we evaluated binary measures of stunting (HAZ less than –2 SD) from age 12 months and overweight (BMIZ greater than 2 SD) from age 6 months,^21^ when these outcomes are more likely to become clinically apparent. Underweight was not evaluated due to a small sample size (present at 525 [3·5%] of 15 011 visits with BMIZ data), including several visits in which five or fewer children who were HEU had underweight.

Covariates of interest were measured at 20–28 weeks’ gestation and included maternal factors, such as age, BMI, educational attainment, perinatal depressive score, intimate partner violence (IPV), and alcohol and tobacco use, and household factors, such as composite score of asset ownership, monthly household income (in ZAR), and parental employment (defined as employment for any parent in the household).^23^ Maternal perinatal depressive symptoms were evaluated using the Edinburgh Postnatal Depression Scale and a score of 13 or greater was used to define depressive symptoms.^24^ Maternal IPV assessment was adapted from the WHO multicountry study^25^ for use in countries in Africa^26^ and was defined as any report of sexual, physical, or emotional violence in the past 12 months. Maternal self-reported tobacco and alcohol use during the 3 months before assessment was assessed using WHO's Alcohol, Smoking and Substance Involvement Screening Test and was dichotomised into any use or no use.^27^ Delivery and postnatal covariates included birthweight, length Z scores,^21^ and breastfeeding status. Self-reported breastfeeding was defined as exclusive breastfeeding for at least 1 month in total versus no breastfeeding or non-exclusive breastfeeding. Study data were collected and managed using REDCap (version 16.0.28).^28^, ^29^

### Statistical analysis

Sample size was based on the number of children with anthropometry data available after birth. No power calculations were done. Recognising that HIV exposure status might affect in-utero growth and postnatal growth differently, the goal of the statistical analysis was to estimate associations between HIV exposure status and anthropometry in each period. We did not publish a statistical analysis plan. We evaluated associations of HIV exposure status with size (birthweight and birth length Z scores) and gestational age (completed weeks and preterm birth status) at birth. We used linear regression to evaluate continuous outcomes and log binomial regression to evaluate binary outcomes. All models were adjusted for covariates measured in pregnancy, including maternal factors (age, BMI, educational attainment, perinatal depressive symptoms, IPV, and alcohol and tobacco use) and household factors (monthly household income, parental employment, and a composite score of asset ownership), and study site.

The primary analysis evaluated associations between HIV exposure status and postnatal growth from age 6 weeks to 8 years. We used linear mixed-effects models with random effects to account for clustering by child and to estimate average WAZ, HAZ, and BMIZ trajectories over time by HIV exposure status. For the secondary outcomes, we used mixed-effects modified Poisson models with a log link and robust-variance estimator to estimate the probability of stunting or overweight by HIV exposure status over time. The same mixed-effects models were used to estimate age-specific and overall (marginalised over time) mean or prevalence estimates and differences by HIV exposure status from age 6 weeks to 8 years. All models included an interaction term for HIV exposure status and time (modelled using an indicator for each visit) to allow associations to vary over time by HIV exposure status. The models were adjusted for the same set of covariates included in the models for size and gestational age at birth, birthweight, and length Z scores (identified using a directed acyclic graph; appendix 1 p 10). Maternal and child postnatal factors, such as diet or physical activity, that influence growth are likely on the causal pathway between HIV exposure status and growth and therefore were not included as covariates. We conducted a sensitivity analysis excluding birthweight and length Z scores.

We used Sankey plots to descriptively evaluate changes in stunting and overweight status over time, both overall and by HIV exposure status. We explored potential effect measure modification by infant sex and exclusive breastfeeding status for at least 1 month in stratified analyses. We conducted exploratory subgroup analyses among children who were HEU of the associations of timing of maternal ART initiation (before *vs* after conception), maternal ART regimen in pregnancy (prophylaxis with zidovudine, efavirenz-based ART with one non-nucleoside reverse transcriptase inhibitor [NNRTI] and two nucleoside reverse transcriptase inhibitors [NRTI], or second-line treatment with a protease inhibitor), and maternal viral load (undetectable [<40 copies per mL] *vs* detectable [≥40 copies per mL]) in pregnancy with anthropometry trajectories. Models among children who were HEU were additionally adjusted for maternal CD4 count. All analyses were conducted using Stata (version 18).

### Role of the funding source

The study sponsors had no role in study design, data collection, data analysis, data interpretation, writing of the report, or the decision to submit for publication.

## Results

**Figure 1:**
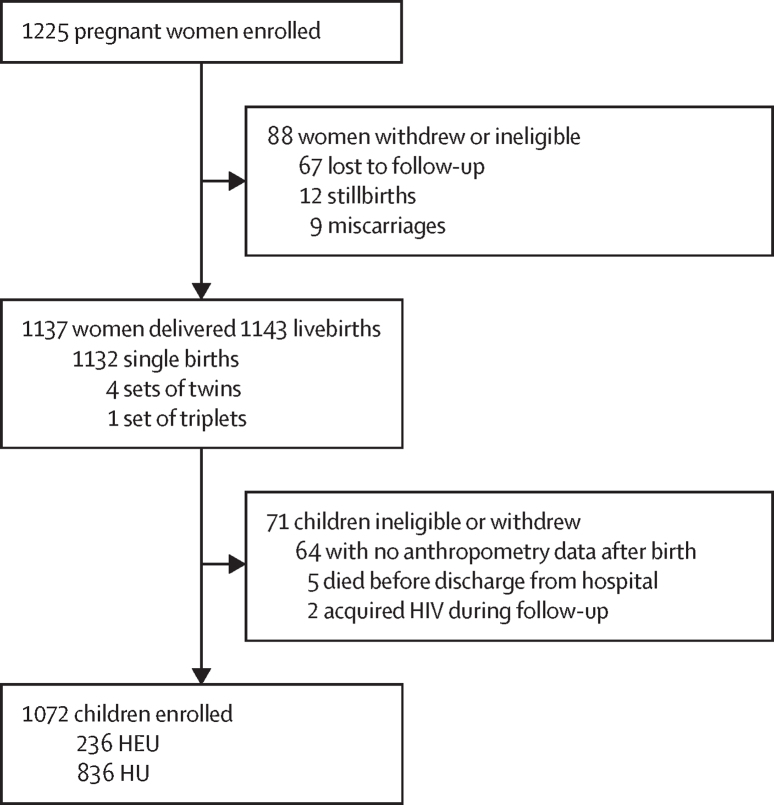
Study population from the Drakenstein Child Health Study HEU=HIV-exposed uninfected. HU=HIV-unexposed.

Between March 5, 2012, and March 31, 2015, 1225 pregnant women were enrolled; 67 (5·5%) mothers were lost to follow-up antenatally, nine (0·7%) had a miscarriage, and 12 (1·0%) had a stillbirth. In total, 1137 women gave birth to 1143 infants (four sets of twins and one set of triplets) from May 29, 2012, to Sept 3, 2015. Of the 1143 livebirths, we excluded from the present analysis five (0·4%) infants who died before discharge from hospital, two (0·2%) children exposed to HIV who were confirmed to have acquired HIV during follow-up, and 64 (5·6%) children who were lost to follow-up and did not have anthropometry data after birth. We did not exclude multiple gestations from the study population. We initially included 1072 (236 [22·0%] HEU and 836 [78·0%] HU; figure 1) children in the study. Children contributed 16 735 visits (3501 [20·9%] HEU and 13 234 [79**·**1%] HU) from age 6 weeks to 8 years (appendix 1 p 4). At cohort enrolment, the mean maternal age was 26**·7** years (SD 5·7) and mean maternal BMI was 28·3 kg/m^2^ (SD 2·0; table 1). Overall, 657 (61·5%) of the 1068 participants in the cohort had lower than a secondary education, 531 (49·6%) of 1070 parents were not currently working, and 925 (86·5%) of 1069 households made less than ZAR5000 (approximately US$450 at the time of the study) per month. 232 (24·3%) of 953 women reported perinatal depressive symptoms, 320 (33·6%) of 953 women reported IPV in the past 12 months, and 303 (28·3%) of 1070 women reported maternal tobacco use. Exclusive breastfeeding for at least 1 month was less common among children who were HEU (82 [35·2%] of 233) than among those who were HU (511 [61·7%] of 828). Among 231 mothers with HIV, 137 (59·3%) initiated ART during the index pregnancy, 185 (80·1%) were on first-line (at the time) efavirenz-based ART, 34 (14·7%) were on zidovudine prophylaxis, and 12 (5·2%) were on second-line protease inhibitor-containing ART. 93 (65·5%) of 142 mothers with viral load data in pregnancy had an undetectable viral load and 75 (39·9%) of 188 mothers with CD4 count data in pregnancy had a CD4 count of at least 500 cells per μL.

**Table 1:** Baseline characteristics of children in the Drakenstein Child Health Study

		**Children who were HEU (n=235)**	**Children who were HU (n=835)**	**Total (N=1070)**
**Characteristics at enrolment at 20–28 weeks’ gestation**				
Maternal age, years		29·6 (5·2)	25·8 (5·5)	26·7 (5·7)
Maternal BMI, kg/m^2^*		29·9 (6·2)	27·8 (6·4)	28·3 (6·4)
Household composite score of asset ownership		6·3 (2·0)	7·0 (2·0)	6·8 (2·0)
Household income in ZAR per month†				
	<1000	83/235 (35·3%)	288/834 (34·5%)	371/1069 (34·7%)
	1000–5000	125/235 (53·2%)	429/834 (51·4%)	554/1069 (51·8%)
	>5000	27/235 (11·5%)	117/834 (14·0%)	144/1069 (13·5%)
Parental employment‡				
	Not working	111 (47·2%)	420 (50·3%)	531 (49·6%)
	Working	124 (52·8%)	415 (49·7%)	539 (50·4%)
Maternal marital status				
	Married or cohabitating	101 (43·0%)	327 (39·2%)	428 (40·0%)
	Single	134 (57·0%)	508 (60·8%)	642 (60·0%)
Maternal education				
	At least secondary or higher	64/234 (27·4%)	347/834 (41·6%)	411/1068 (38·5%)
	Lower than secondary	170/234 (72·6%)	487/834 (58·4%)	657/1068 (61·5%)
Maternal perinatal depressive symptoms§		53/206 (25·7%)	179/747 (24·0%)	232/953 (24·3%)
Any maternal intimate partner violence in the past 12 months¶		58/206 (28·2%)	262/747 (35·1%)	320/953 (33·6%)
Any maternal alcohol use‖				
	No use	203/226 (89·8%)	685/794 (86·3%)	888/1020 (87·1%)
	Any use	23/226 (10·2%)	109/794 (13·7%)	132/1020 (12·9%)
Any maternal tobacco use‖		29 (12·3%)	274 (32·8%)	303 (28·3%)
Site				
	Mbekweni	219 (93·2%)	373 (44·7%)	592 (55·3%)
	TC Newman	16 (6·8%)	462 (55·3%)	478 (44·7%)
**Delivery and postnatal characteristics**				
Infant sex				
	Female	107 (45·5%)	416 (49·8%)	523 (48·9%)
	Male	128 (54·5%)	419 (50·2%)	547 (51·1%)
Exclusive breastfeeding for >1 month				
	Yes	82/233 (35·2%)	511/828 (61·7%)	593/1061 (55·9%)
	No	151/233 (64·8%)	317/828 (38·3%)	468/1061 (44·1%)
Ever exclusively breastfed				
	Yes	106 (45·1%)	758 (90·8%)	864 (80·7%)
	No	129 (54·9%)	77 (9·2%)	206 (19·3%)
Duration of exclusive breastfeeding, months				
	Median (IQR)	0 (0–2·8)	1·8 (0·9–3·2)	1·5 (0·7–3·2)
	Range	0–7	0–8	0–8

Data are n (%), n/N (%), and mean (SD) unless otherwise stated. Two infants (0·2%; one HEU and one HU) were missing information at birth and were excluded from this table. There were missing data for the 1070 children in the categories of maternal education (n=2 [0·2%]), household income (n=1 [0·1%]), maternal perinatal depression (n=117 [10·9%]), maternal intimate partner violence (n=117 [10·9%]), maternal prenatal alcohol exposure (n=50 [4·7%]), and maternal BMI (n=33 [3·1%]). HEU=HIV-exposed uninfected. HU=HIV-unexposed.

* 227 (96·7%) of 235 children who were HEU and 810 (97·0%) of 835 children who were HU had maternal BMI data available.

† <ZAR1000 is equivalent to <US$90 and >ZAR5000 is equivalent to >$450 at the time of the study.

‡ Parental employment was defined as yes if any parent present in the household was working.

§ Measured with the Edinburgh Depression Scale: ≥13 indicates depressive symptoms.

¶ Includes any physical, emotional, or sexual violence.

‖ Based on self-report in the Alcohol, Smoking, and Substance Involvement Screening Test.

At delivery, the mean birthweight was 3035 g (SD 592; children who were HEU 3012 g [598] *vs* children who were HU 3041 g [590]), the mean birthweight Z score was –0·28 (SD 1·05; children who were HEU –0·26 [1·01] *vs* children who were HU –0·28 [1·06]), and the mean birth length Z score was 0·42 (SD 1·60; children who were HEU 0·32 [1·64] *vs* children who were HU 0·45 [1·59]; table 2). Overall, 168 (15·7%) of 1070 infants were preterm (43 [18·3%] of 235 children who were HEU *vs* 125 [15·0%] of 835 children who were HU). In multivariable analyses, there were no differences in size, gestational age, or preterm birth status by HIV exposure status, although differences in birth length Z scores were larger for children who were HEU than children who were HU after adjustment (mean difference –0·26 (95% CI –0·54 to 0·01; table 2).

**Table 2:** Associations between HIV exposure status and size and gestational age at birth

		**Total (N=1070)**	**Children who were HEU (n=235)**	**Children who were HU (n=835)**	**Unadjusted mean difference (95% CI)**	**Adjusted mean difference (95% CI)**
Birthweight, g		3035 (592)	3012 (598)	3041 (590)	−29·6 (−115·3 to 56·1)	−81·3 (−178·7 to 16·1)
	Female	3014 (587)	2953 (635)	3029 (574)	..	..
	Male	3055 (596)	3060 (564)	3053 (607)	..	..
Birthweight Z score		−0·28 (1·05)	−0·26 (1·01)	−0·28 (1·06)	0·02 (−0·14 to 0·17)	−0·14 (−0·31 to 0·04)
Birth length Z score		0·42 (1·60)	0·32 (1·64)	0·45 (1·59)	−0·12 (−0·36 to 0·11)	−0·26 (−0·54 to 0·01)
Gestational age at delivery, weeks		38 (2·4)	38 (2·6)	38 (2·4)	−0·2 (−0·6 to 0·1)	−0·1 (−0·5 to 0·3)
Preterm birth*		168 (15·7%)	43 (18·3%)	125 (15·0%)	1·22 (0·89 to 1·67)†	1·23 (0·82 to 1·86)†

Data are mean (SD) or n (%) unless otherwise stated. Two infants (0·2%; one HEU and one HU) were missing information at birth. Adjusted models were adjusted for maternal education, household composite score of asset ownership, maternal BMI, maternal age, maternal perinatal depressive symptoms, maternal intimate partner violence, any maternal alcohol use, maternal smoking at cohort enrolment, parental employment, household income, and study site. Mean differences and risk ratios compare children who were HEU to children who were HU. HEU=HIV-exposed uninfected. HU=HIV-unexposed.

* Defined as delivery <37 weeks’ gestation.

† Risk ratio (95% CI).

Mean overall WAZ were –0·27 (SD 1·18) at age 6–10 weeks, increasing to 0·18 (1·29) at 9 months and declining to –0·31 (1·28) at 8 years (appendix 1 p 5). Similarly, overall BMIZ were 0·15 (SD 1·14) at age 6–10 weeks, increasing to 0·88 (1·58) at 18 months and declining to –0·17 (1·24) at 8 years (appendix 1 p 7). Conversely, overall mean HAZ were –0·72 (SD 1·31) at age 6 weeks, were lowest at 36 months (–1·14 [1·14]), and then increased, but stayed lower than the WHO reference mean of 0 throughout until 8 years (–0·35 [1·05]; appendix 1 p 6).

**Figure 2:**
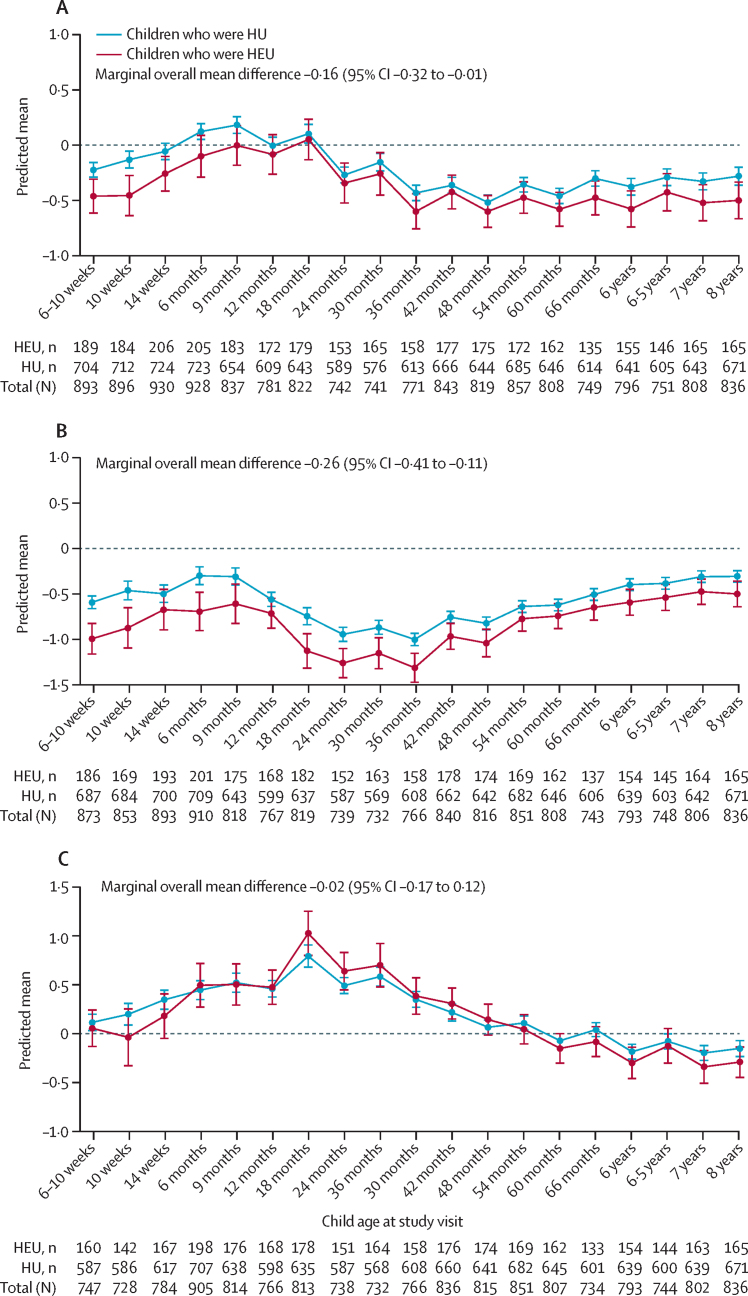
WAZ, HAZ, and BMIZ by HIV exposure status from age 6 weeks to 8 years Predicted mean WAZ (A), HAZ (B), and BMIZ (C) over time by HIV exposure status. Error bars represent 95% CI. Predicted means and marginal overall mean differences come from a linear mixed-effects model adjusted for clustering by child and for parental employment, household income, maternal education, household asset score, maternal BMI, maternal age, maternal perinatal depressive score, maternal intimate partner violence, maternal alcohol use, maternal smoking at cohort enrolment, birthweight Z score, birth length Z score, and study site. BMIZ=BMI-for-age Z score. HAZ=height-for-age Z score. HEU=HIV-exposed uninfected. HU=HIV-unexposed. WAZ=weight-for-age Z score.

In multivariable analyses, children who were HEU overall had lower WAZ than children who were HU (marginal mean difference –0·16 [95% CI –0·32 to –0·01]); the largest differences were observed in the first 6 months of life (–0·23 [–0·44 to –0·01]; appendix 1 p 5), after which differences by HIV exposure status were smaller but WAZ remained lower in children who were HEU than children who were HU (figure 2). Children who were HEU also had lower overall HAZ (–0·26 [–0·41 to –0·11]) than children who were HU, with the largest differences in the first 3 years of life (–0·33 [–0·52 to –0·13]; appendix 1 p 6), after which differences in HAZ were smaller but children who were HEU had lower HAZ than children who were HU through to age 8 years. No evidence of differences in BMIZ by HIV exposure status from age 6 weeks to 8 years was found (–0·02 [–0·17 to 0·12]). Results were similar when birthweight and length Z scores were excluded in sensitivity analyses (appendix 1 p 11).

**Figure 3:**
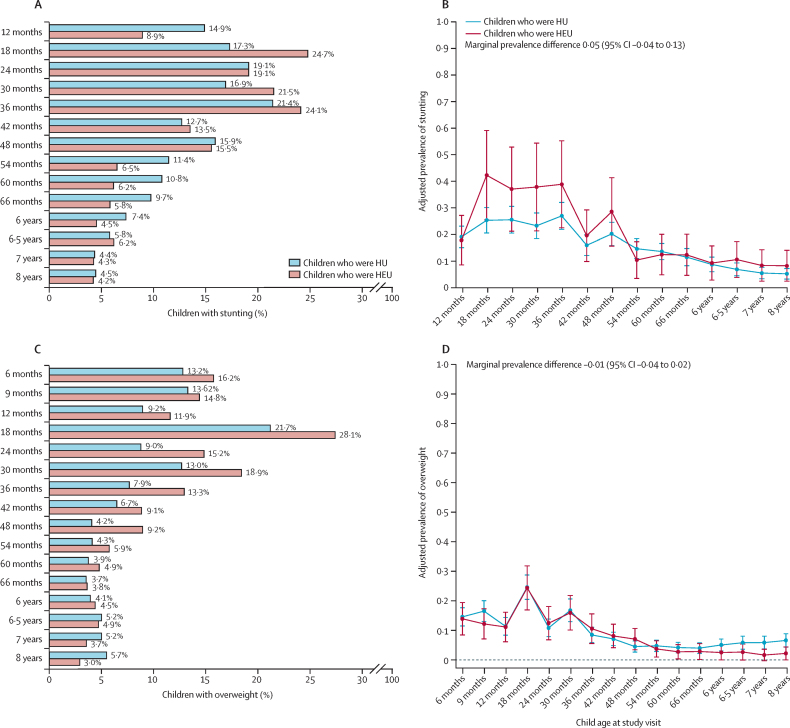
Prevalence of stunting and overweight by HIV exposure status and age (A) Unadjusted prevalence of stunting (height-for-age Z score <–2 SD) by HIV exposure status and age. (B) Adjusted prevalence of stunting over time by HIV exposure status; error bars represent 95% CI. (C) Unadjusted prevalence of overweight (BMI-for-age Z score >2 SD) by HIV exposure status and age. (D) Adjusted prevalence of overweight over time by HIV exposure status; error bars represent 95% CI. Predicted prevalence at individual timepoints by HIV exposure status and marginal overall prevalence differences come from a Poisson mixed-effects model with a log link and robust-variance estimator adjusted for clustering by child and for parental employment, household income, maternal education, household asset score, maternal BMI, maternal age, maternal perinatal depressive score, maternal intimate partner violence, maternal alcohol use, maternal smoking at cohort enrolment, birthweight Z score, birth length Z score, and study site. HEU=HIV-exposed uninfected. HU=HIV-unexposed.

In the cohort, the unadjusted prevalence of stunting peaked at age 3 years (168 [21·9%] of 766; appendix 1 p 8) and then declined to less than 5% by age 8 years for both children who were HU and children who were HEU (figure 3). In multivariable analyses, there was no evidence that HIV exposure status was associated with stunting from age 12 months to 8 years (marginal prevalence difference 0·05 [95% CI –0·04 to 0·13]). Most changes in stunting status occurred before age 5 years, with few differences by HIV exposure status (appendix 1 p 15).

The unadjusted prevalence of being overweight was highest at age 18 months (188 [23·1%] of 813; appendix 1 p 9) and then declined until 8 years to 3·0% (five of 165) for children who were HEU and 5·7% (38 of 671) for children who were HU (figure 3). In multivariable analyses, HIV exposure status was not associated with being overweight from age 6 months to 8 years (marginal prevalence difference –0·01 [95% CI –0·04 to 0·02]). Changes in overweight status occurred predominantly before age 3 years, with few differences by HIV exposure status (appendix 1 p 15).

In multivariable stratified analyses exploring sex as a potential modifier, associations of HIV exposure status with WAZ and HAZ were numerically larger among male children than female children (appendix 1 p 12) but confidence intervals were overlapping. Similarly, associations of HIV exposure status with WAZ and HAZ were numerically larger in children not exclusively breastfed for at least 1 month compared with children exclusively breastfed for at least 1 month (appendix 1 p 13), but confidence intervals overlapped. In subgroup analyses restricted to children who were HEU, there was little evidence of associations between timing of maternal ART initiation, maternal ART regimen, or maternal viral load in pregnancy and anthropometry outcomes (appendix 1 p 14).

## Discussion

In this cohort of South African children followed up longitudinally, we did not observe differences in size or gestational age at birth by HIV exposure status. Postnatally, children aged 6 weeks to 8 years who were HEU had lower overall HAZ and WAZ, but no difference in BMIZ, than did children who were HU. Whereas the largest differences between children who were HEU and HU for WAZ were observed in the first 6 months of life, for HAZ, the largest differences were noted in the first 3 years of life. WAZ and HAZ were often lower than WHO reference means, regardless of HIV exposure. Lower WAZ and HAZ among children who were HEU than among those who were HU did not translate into an increased prevalence of stunting or being overweight until age 8 years. The prevalence of stunting and being overweight declined for both children who were HEU and HU over time to 6% or less by age 8 years. In exploratory analyses among children who were HEU with near-universal ART exposure in gestation, there was little evidence that maternal HIV or ART factors were associated with anthropometry Z scores from age 6 weeks to 8 years.

Within the DCHS cohort, there were no statistical differences in size or gestational age at birth between children who were HEU and HU. However, children who were HEU had a mean birth length Z score 0·26 SD (95% CI –0·54 to 0·01) smaller than children who were HU after adjustment for covariates, which could be clinically meaningful. Smaller size, particularly length, at birth has been observed in other cohorts when comparing children who are HEU with those who are HU,^6^, ^30^ indicating that impaired growth among children who are HEU might begin during gestation and be extended or exacerbated postnatally. Understanding differences in drivers of fetal and postnatal growth for these children is essential to support future intervention development, which should consider both fetal and postnatal growth.

Our finding that children who were HEU had lower HAZ and WAZ than children who were HU provides some of the first longitudinal evidence of sustained growth deficits for children who are HEU until age 8 years. Lower HAZ within the first 2 years of life in children who are HEU than in those who are HU is well documented in a 2025 meta-analysis,^3^ with considerably less available evidence beyond age 2 years.^14^ We observed the largest differences in WAZ and HAZ in early childhood after adjustment for birth size and other risk factors during pregnancy. Although differences in HAZ and WAZ became smaller after early childhood with and without HIV exposure, the scores for children who were HEU remained lower than for children who were HU until age 8 years, indicating that early-life growth deficits persist into childhood. Given that children in the DCHS did not differ statistically in size at birth, these findings also suggest that the largest deficits in growth among children who are HEU emerge postnatally in early childhood. Growth faltering in early life is associated with increased risk of cardiometabolic disease later in life,^4^ suggesting that these children might be at increased risk of cardiometabolic comorbidities in adulthood compared with children who are HU. To better understand the implications of early-life growth deficits in children who are HEU, a clearer understanding of early-life drivers of growth, such as increased infection burden,^9^ is needed.

When evaluating differences in postnatal growth between children who are HEU and HU, it is important to consider that all children within the DCHS cohort mainly had WAZ and HAZ lower than mean WHO values through to age 8 years. As has been reported in other South African cohorts,^31^ HAZ values were approximately 0·5–1·0 standard deviations lower than the WHO reference mean from age 6 weeks to 8 years for children who were HEU or HU. These findings suggest that nutritional deficiencies and other drivers of suboptimal growth in this population persist regardless of HIV exposure and that deficits in HAZ and WAZ for children who are HEU might be even larger when compared with children who are HU with Z scores closer to mean WHO values.

Exploratory analyses suggested few differences in anthropometry by maternal HIV or ART characteristics in this cohort, with high ART coverage and viral load suppression during pregnancy but a short breastfeeding duration.^10^, ^11^ Although subgroup analyses to detect associations between maternal HIV and ART characteristics with anthropometry outcomes were likely underpowered, very large cohorts would be needed for well powered analyses. Future efforts to combine and do meta-analyses of data would help to clarify the contribution of HIV infection, relative to ART treatment, on in-utero and postnatal growth for children who are HEU. The DCHS cohort was enrolled between 2012 and 2015, when first-line therapy was efavirenz-based ART. First-line HIV treatment in South Africa now includes dolutegravir, an integrase strand inhibitor that has been associated with weight gain in adults. Current evidence suggests that length or height might be improved in children who are HEU and exposed to dolutegravir versus efavirenz,^32^ but growth deficits relative to children who are HU remain in the context of dolutegravir exposure.^33^

Our analysis has strengths and limitations. Strengths include the ability to examine longitudinal anthropometry trajectories in a cohort of children who are HEU and HU with little loss to follow-up, with adjustment for psychosocial variables that might influence growth not always included in analyses.^6^, ^10^, ^12^ We were also able to explore potential postnatal modifiers of growth and, among children who were HEU, whether maternal HIV and ART was associated with anthropometry trajectories. Limitations include the exclusion of BMIZ before 50 weeks’ gestation for infants born preterm, as they are not defined, which could make BMIZ results within the first year more generalisable to term infants. We did not examine additional indicators of anthropometry—such as head circumference or body composition—or indicators of growth, such as catch-up growth or growth velocity. Focusing on anthropometry Z scores alone does not account for the increasing width of single Z score measures as children grow up. Therefore, additional approaches, including height-for-age deficit, would be required to explore how much of an absolute deficit in height is maintained in children who are HEU compared with children who are HU.^34^ We did not conduct mediation analyses to understand how postnatal maternal or child covariates could affect the relationship between in-utero HIV exposure status and postnatal child growth. The short duration of breastfeeding within South Africa might limit the generalisability of findings to high HIV burden settings with a longer duration of breastfeeding. Finally, binary measures of stunting or overweight might not include all children affected by a suboptimal growth environment.

In a South African cohort with similar size and gestational age at birth, we observed small deficits in WAZ and HAZ, but not BMIZ, stunting, or overweight among children who were HEU from age 6 weeks to 8 years. Differences in WAZ and HAZ in children who were HEU compared with HU emerged postnatally and were largest in early childhood. Until age 8 years, mean WAZ and HAZ were lower than the WHO reference mean for children who were HEU or HU, indicating that interventions to optimise growth for all children within this setting remain crucial. Early-life growth deficits remain predictors of adverse health outcomes in later life. Future work should focus on understanding the aetiology and clinical implications of early-life growth deficits among children who are HEU to optimise health outcomes.

### Contributors

### Equitable Partnership Declaration

### Data sharing

The Drakenstein Child Health Study is committed to the principle of data sharing. De-identified data will be made available to requesting researchers as appropriate. Requests for collaborations are welcome. More information can be found on our website.

## Declaration of interests

We declare no competing interests.

## Supplementary Materials

Supplementary appendix 1

Equitable Partnership Declaration
