# Supplementary material for: In-utero HIV exposure and postnatal growth to 8 years of age: a prospective cohort study in the Western Cape, South Africa
Source: Lancet Child Adolesc Health. 2026 Sep;10(9):658–67. doi: 10.1016/S2352-4642(26)00121-5 (PMC13437777; doi:10.1016/S2352-4642(26)00121-5)
Supplement: Supplementary file 1 [file mmc1.pdf]

THE LANCET
Child & Adolescent Health

Supplementary appendix 1

This appendix formed part of the original submission and has been peer reviewed. We post it as supplied by the authors.

Supplement to: Bengtson AM, Pellowski JA, Botha M, et al. In-utero HIV exposure and postnatal growth to 8 years of age: a prospective cohort study in the Western Cape, South Africa. *Lancet Child Adolesc Health* 2026; published online July 17. https://doi.org/10.1016/S2352-4642(26)00121-5.

Webappendix

Contents

Additional cohort information 2

Rationale for including multiples 2

Additional covariate information 2

Rationale for using BMIZ scores in children less than 2 years of age 2

Implausible Z-scores 3

Table S1. Study population at each study visit by HIV exposure status 4

Table S2. Weight-for-age-z-scores at individual time points overall and by HIV exposure status. 5

Table S3. Height-for-age-z-scores at individual time points overall and by HIV exposure status. 6

Table S4. Body mass index-for-age-z-scores at individual time points overall and by HIV exposure status. 7

Table S5. Stunting (height-for-age-z-score <-2) at individual time points overall and by HIV exposure status. 8

Table S6. Overweight (body mass index-for-age-z-scores > 2) at individual time points overall and by HIV exposure status. 9

Figure S1. Directed Acyclic Graph. 10

Figure S2. Weight for Age (WAZ), Height for Age (HAZ) and Body Mass Index (BMIZ) scores by HIV exposure status from 6 weeks to 8 years of age, excluding covariates measured at birth. 11

Figure S3. Weight for Age (WAZ), Height for Age (HAZ) and Body Mass Index (BMIZ) scores stratified by infant sex. 12

Figure S4. Weight for Age (WAZ), Height for Age (HAZ) and Body Mass Index (BMIZ) scores stratified by exclusive breastfeeding status (EBF). 13

Figure S5. Subgroup analyses of Weight for Age (WAZ), Height for Age (HAZ) and Body Mass Index (BMIZ) scores among HIV exposed, but uninfected children 14

Figure S6. Sankey plot of change in stunting (HAZ < -2SD) and overweight (BMIZ > 2 SD) status by age and HIV exposure status. 15

References 16

Additional cohort information

The prevalence of HIV among women attending antenatal care in the Westen Cape Province was 20.5% in 2012, the year that enrollment into the Drakenstein Child Health Study (DCHS) began.(1) Characteristics of HIV infection and ART use at enrollment into the cohort have been published previously.(2) Briefly, of 1225 women enrolled in the study, 260 (21%) women were living with HIV. Among women living with HIV, 182 (71%) were diagnosed before pregnancy and 72 (28%) were diagnosed in pregnancy. Information on maternal CD4 count and viral load during pregnancy was accessed from the online National Health Laboratory Service system or maternal self-report. If more than one result for CD4 count or viral load was available during pregnancy, the result closest to birth was used. Two children born to women living with HIV acquired HIV during the follow-up period; one at 4 months and one at 18 months. (2)

Information on the inclusion criteria for the DCHS and their rationale have been previously published.(3) Briefly, women enrolled were required to be at least 18 years of age because women under 18 years of age cannot provide consent for their infant. Women were enrolled from 20-28 weeks gestation, during the second trimester of pregnancy with the goal of enrolling a study population generalizable to pregnant women in South Africa. At the time of the study, 60% of women in the Western Cape (where the study sites are located) presented for their first antenatal care visit in the second trimester and the mean gestational age at first antenatal care visit was 23 weeks (SD 7.2).(4)

Rationale for including multiples

The final study population included 4 sets of twins and 2 children who were born as set of triplets (n=10/1072, 0.9%). Twins and triplets are well known to grow more slowly in utero and to be smaller at birth than singleton fetuses.(5) However, multiples typically catch up in growth to singleton infants in length and weight as early as 3 months of age.(6, 7) Given our interest in examining postnatal growth through 8 years of age, we did not exclude multiples from our study population.

Additional covariate information

Gestational age at birth was estimated based on an ultrasound done in the second trimester.(8) If ultrasound was unavailable then symphysis-fundal height, recorded by trained clinical staff at enrolment, or last menstrual period was used. Information on pregnancy complications, including pre-eclampsia, eclampsia, hypertension in pregnancy, and gestational diabetes were collected by study staff during pregnancy and via chart review. However, prevalence of pregnancy complications was low (<5%), thus were not considered in this analysis.(8)

Rationale for using BMIZ scores in children less than 2 years of age

As has been done previously, we chose to use BMIZ in children less than 2 years of age, rather than weight-for-length or height z-scores (WFHZ) as BMIZ is more comparable to measures of adiposity in childhood (9) and to ensure that measures were comparable in measures across the follow-up period (6

weeks to 8 years of age) within DCHS. We evaluated correlation between using BMIZ and WFHZ to identify overweight children (BMIZ or WFHZ>2SD) at 2 years of age. We observed very strong correlation between the two measures (>96% concordance between the measures; Cramér's V: 0.84), suggesting that BMIZ are comparable to WFHZ within this cohort and can be used to identify overweight status in children less than 2 years of age.

wfhz_cat2v2	*bmiz_cat2*		***Total***
	Overweight	Unlikely overweight	
Overweight	86<br>98.9 %	1<br>1.1 %	87<br>100 %
Unlikely overweight	30<br>3.7 %	780<br>96.3 %	810<br>100 %
Total	116<br>12.9 %	781<br>87.1 %	897<br>100 %

χ^2=623.228 · df=1 · φ=0.839 · p=0.000

Implausible Z-scores

Implausible values for anthropometry z-scores were identified based on WHO recommendations and excluded from analyses.(10) Implausible z-scores were defined as HAZ (<-6 or >+6) , WAZ (<-6 or >+5), and BMIZ (<-5 or >+5) based on WHO guidance. (10) We excluded individual implausible z-score observations, not all observations for a given participant. In accordance with WHO recommendations, we report on the portion of children who had an observation excluded due to being implausible. Two (both <-6.0_ HAZ observations were excluded (2/1072 participants = 0.2%). Six (2 <-6.0 and 4 >+6.0) BMIZ observations were excluded due to being implausible (6/1072 participants=0.6%).

Table S1. Study population at each study visit by HIV exposure status

	HIV exposed, uninfected n (%)	HIV unexposed n (%)	Total N
Birth	235 (22%)	835 (78%)	1,070
6-10 weeks	189 (21%)	705 (79%)	894
10 weeks	186 (20%)	723 (80%)	909
14 weeks	209 (22%)	727 (78%)	936
6 months	208 (22%)	725 (78%)	933
9 months	183 (22%)	657 (78%)	840
12 months	172 (22%)	609 (78%)	781
18 months	183 (22%)	644 (78%)	827
24 months	154 (21%)	589 (79%)	743
30 months	165 (22%)	576 (78%)	741
36 months	158 (20%)	613 (80%)	771
42 months	179 (21%)	668 (79%)	847
48 months	175 (21%)	645 (79%)	820
54 months	172 (20%)	685 (80%)	857
60 months	162 (20%)	650 (80%)	812
66 months	139 (18%)	619 (82%)	758
6 years	155 (19%)	641 (81%)	796
6.5 years	147 (19%)	608 (81%)	755
7 years	165 (20%)	644 (80%)	809
8 years	165 (20%)	671 (80%)	836
Total Visits	3501 (21%)	13234 (79%)	16,735

Table S2. Weight-for-age-z-scores at individual time points overall and by HIV exposure status.

	**Overall** N=1070 Unadjusted Mean (SD)	**Children HIV exposed uninfected** n=235 Adjusted Mean 95% CI*	**Children HIV unexposed** n=835 Adjusted Mean 95% CI*	Adjusted Mean Difference* (95% CI)
6-10 weeks	-0.27(1.18)	-0.22 (-0.30 - -0.15)	-0.47 (-0.63 - -0.30)	-0.24 (-0.42--0.06)
10 weeks	-0.18(1.25)	-0.13 (-0.21 - -0.05)	-0.46 (-0.65 - -0.27)	-0.33 (-0.54--0.12)
14 weeks	-0.09(1.22)	-0.05 (-0.14 - 0.03)	-0.26 (-0.43 - -0.09)	-0.21 (-0.39--0.02)
6 months	0.08(1.27)	0.13 (0.05 - 0.21)	-0.10 (-0.30 - 0.10)	-0.23 (-0.44--0.01)
9 months	0.18(1.29)	0.19 (0.10 - 0.27)	0.00 (-0.19 - 0.19)	-0.19 (-0.40-0.02)
12 months	-0.02(1.29)	0.00 (-0.09 - 0.08)	-0.08 (-0.27 - 0.11)	-0.08 (-0.29-0.13)
18 months	0.11(1.32)	0.11 (0.02 - 0.20)	0.06 (-0.14 - 0.25)	-0.05 (-0.27-0.16)
24 months	-0.29(1.23)	-0.27 (-0.35 - -0.19)	-0.35 (-0.54 - -0.16)	-0.07 (-0.28-0.13)
30 months	-0.18(1.24)	-0.15 (-0.24 - -0.07)	-0.26 (-0.46 - -0.06)	-0.11 (-0.33-0.11)
36 months	-0.46(1.15)	-0.44 (-0.51 - -0.36)	-0.61 (-0.77 - -0.44)	-0.17 (-0.36-0.02)
42 months	-0.36(1.15)	-0.37 (-0.45 - -0.29)	-0.43 (-0.59 - -0.27)	-0.06 (-0.24-0.12)
48 months	-0.53(1.10)	-0.52 (-0.60 - -0.45)	-0.61 (-0.76 - -0.46)	-0.08 (-0.26-0.09)
54 months	-0.38(1.08)	-0.36 (-0.43 - -0.29)	-0.48 (-0.63 - -0.33)	-0.12 (-0.29-0.05)
60 months	-0.49(1.16)	-0.47 (-0.54 - -0.39)	-0.59 (-0.75 - -0.43)	-0.12 (-0.30-0.06)
66 months	-0.36(1.15)	-0.30 (-0.38 - -0.23)	-0.48 (-0.64 - -0.32)	-0.18 (-0.36-0.00)
6 years	-0.44(1.21)	-0.38 (-0.46 - -0.30)	-0.58 (-0.76 - -0.41)	-0.20 (-0.4--0.01)
6.5 years	-0.31(1.23)	-0.29 (-0.38 - -0.21)	-0.43 (-0.61 - -0.25)	-0.14 (-0.34-0.06)
7 years	-0.35(1.25)	-0.33 (-0.41 - -0.25)	-0.53 (-0.70 - -0.36)	-0.20 (-0.39-0.00)
8 years	-0.31(1.28)	-0.28 (-0.70 - -0.36)	-0.51 (-0.68 - -0.33)	-0.22 (-0.42--0.02)

*Adjusted estimates come from a linear mixed effects model adjusted for clustering by child and for parental employment, household income, maternal education, household asset score, maternal BMI, maternal age, maternal depression, maternal intimate partner violence, maternal alcohol use, maternal smoking at cohort enrollment, birthweight zscore, birth length zscore, and site. Mean differences compare children exposed to HIV, but uninfected to children HIV unexposed.

Table S3. Height-for-age-z-scores at individual time points overall and by HIV exposure status.

	**Overall** N=1070 Unadjusted Mean (SD)	**Children HIV unexposed** n=235 Adjusted Mean (95% CI*)	**Children HIV exposed uninfected** n=835 Adjusted Mean (95% CI*)	Adjusted Mean Difference* (95% CI)
6-10 weeks	-0.72(1.31)	-0.62 (-0.70 - -0.54)	-1.05 (-1.23 - -0.86)	-0.42 (-0.63--0.22)
10 weeks	-0.58(1.65)	-0.49 (-0.60 - -0.37)	-0.92 (-1.16 - -0.68)	-0.43 (-0.70--0.16)
14 weeks	-0.58(1.61)	-0.52 (-0.63 - -0.42)	-0.71 (-0.95 - -0.47)	-0.19 (-0.45-0.08)
6 months	-0.42(1.58)	-0.32 (-0.43 - -0.21)	-0.73 (-0.96 - -0.50)	-0.41 (-0.67--0.16)
9 months	-0.37(1.50)	-0.33 (-0.43 - -0.22)	-0.64 (-0.88 - -0.40)	-0.32 (-0.58--0.06)
12 months	-0.62(1.26)	-0.59 (-0.68 - -0.50)	-0.75 (-0.93 - -0.57)	-0.16 (-0.36-0.04)
18 months	-0.86(1.41)	-0.79 (-0.89 - -0.68)	-1.19 (-1.40 - -0.98)	-0.40 (-0.64--0.17)
24 months	-1.06(1.19)	-1.00 (-1.08 - -0.91)	-1.33 (-1.51 - -1.15)	-0.33 (-0.53--0.13)
30 months	-0.95(1.18)	-0.91 (-1.00 - -0.83)	-1.22 (-1.40 - -1.03)	-0.30 (-0.51--0.09)
36 months	-1.14(1.14)	-1.06 (-1.13 - -0.98)	-1.38 (-1.56 - -1.21)	-0.33 (-0.52--0.13)
42 months	-0.85(1.08)	-0.80 (-0.87 - -0.72)	-1.02 (-1.18 - -0.86)	-0.22 (-0.40--0.04)
48 months	-0.92(1.12)	-0.87 (-0.95 - -0.79)	-1.10 (-1.26 - -0.93)	-0.23 (-0.42--0.04)
54 months	-0.71(1.06)	-0.67 (-0.75 - -0.60)	-0.81 (-0.97 - -0.66)	-0.14 (-0.31-0.03)
60 months	-0.68(1.05)	-0.66 (-0.73 - -0.58)	-0.78 (-0.94 - -0.63)	-0.13 (-0.30-0.05)
66 months	-0.57(1.07)	-0.53 (-0.61 - -0.46)	-0.68 (-0.84 - -0.53)	-0.15 (-0.33-0.03)
6 years	-0.47(1.05)	-0.42 (-0.49 - -0.34)	-0.62 (-0.78 - -0.47)	-0.20 (-0.38--0.03)
6.5 years	-0.42(1.07)	-0.40 (-0.48 - -0.33)	-0.57 (-0.73 - -0.41)	-0.16 (-0.34-0.02)
7 years	-0.35(1.05)	-0.33 (-0.40 - -0.25)	-0.50 (-0.65 - -0.35)	-0.17 (-0.35-0.00)
8 years	-0.35(1.05)	-0.32 (-0.65 - -0.35)	-0.53 (-0.68 - -0.37)	-0.20 (-0.38--0.03)

*Adjusted estimates come from a linear mixed effects model adjusted for clustering by child and for parental employment, household income, maternal education, household asset score, maternal BMI, maternal age, maternal depression, maternal intimate partner violence, maternal alcohol use, maternal smoking at cohort enrollment, birthweight zscore, birth length zscore, and site. Mean differences compare children exposed to HIV, but uninfected to children HIV unexposed.

Table S4. Body mass index-for-age-z-scores at individual time points overall and by HIV exposure status.

	**Overall** N=1070	**Children HIV unexposed** n=235	**Children HIV exposed uninfected** n=835	
	Unadjusted Mean (SD)	Adjusted Mean 95% CI*	Adjusted Mean 95% CI*	Adjusted Mean Difference* (95% CI)
6-10 weeks	0.15(1.14)	0.12 (0.03 - 0.22)	0.06 (-0.14 - 0.26)	-0.06 (-0.29-0.16)
10 weeks	0.21(1.5)	0.21 (0.09 - 0.33)	-0.04 (-0.34 - 0.27)	-0.24 (-0.57-0.09)
14 weeks	0.35(1.35)	0.36 (0.25 - 0.47)	0.19 (-0.05 - 0.43)	-0.17 (-0.44-0.09)
6 months	0.50(1.45)	0.46 (0.36 - 0.57)	0.51 (0.28 - 0.75)	0.05 (-0.21-0.31)
9 months	0.54(1.4)	0.54 (0.43 - 0.65)	0.52 (0.30 - 0.75)	-0.02 (-0.27-0.23)
12 months	0.46(1.24)	0.48 (0.38 - 0.57)	0.49 (0.30 - 0.68)	0.02 (-0.20-0.23)
18 months	0.88(1.58)	0.82 (0.70 - 0.94)	1.06 (0.82 - 1.30)	0.24 (-0.03-0.51)
24 months	0.51(1.24)	0.51 (0.42 - 0.60)	0.66 (0.46 - 0.87)	0.15 (-0.07-0.38)
30 months	0.60(1.38)	0.61 (0.50 - 0.71)	0.73 (0.49 - 0.96)	0.12 (-0.14-0.38)
36 months	0.39(1.21)	0.36 (0.27 - 0.45)	0.40 (0.20 - 0.60)	0.04 (-0.18-0.26)
42 months	0.28(1.23)	0.23 (0.13 - 0.32)	0.32 (0.15 - 0.49)	0.09 (-0.11-0.29)
48 months	0.10(1.13)	0.07 (-0.01 - 0.15)	0.15 (-0.02 - 0.32)	0.08 (-0.11-0.27)
54 months	0.11(1.12)	0.12 (0.03 - 0.20)	0.05 (-0.11 - 0.21)	-0.07 (-0.25-0.12)
60 months	-0.09(1.12)	-0.07 (-0.15 - 0.01)	-0.15 (-0.31 - 0.01)	-0.08 (-0.27-0.10)
66 months	-0.01(1.11)	0.05 (-0.04 - 0.13)	-0.08 (-0.25 - 0.08)	-0.13 (-0.31-0.06)
6 years	-0.23(1.17)	-0.19 (-0.27 - -0.10)	-0.31 (-0.48 - -0.13)	-0.12 (-0.31-0.08)
6.5 years	-0.09(1.18)	-0.08 (-0.17 - 0.01)	-0.13 (-0.32 - 0.06)	-0.05 (-0.26-0.17)
7 years	-0.22(1.20)	-0.20 (-0.29 - -0.12)	-0.35 (-0.53 - -0.17)	-0.15 (-0.35-0.06)
8 years	-0.17(1.24)	-0.15 (-0.53 - -0.17)	-0.30 (-0.46 - -0.13)	-0.14 (-0.34-0.05)

*Adjusted estimates come from a linear mixed effects model adjusted for clustering by child and for parental employment, household income, maternal education, household asset score, maternal BMI, maternal age, maternal depression, maternal intimate partner violence, maternal alcohol use, maternal smoking at cohort enrollment, birthweight zscore, birth length zscore, and site. Mean differences compare children exposed to HIV, but uninfected to children HIV unexposed.

Table S5. Stunting (height-for-age-z-score <-2) at individual time points overall and by HIV exposure status.

	**Overall** N=1070 Unadjusted n (%)	**Children HIV unexposed** n=235 Adjusted Prevalence 95% CI*	**Children HIV exposed uninfected** n=835 Adjusted Prevalence 95% CI*	Adjusted Prevalence Difference* (95% CI)
12 months	104 (13.6)	0.19 (0.15 - 0.23)	0.18 (0.08 - 0.27)	-0.01 (-0.11-0.09)
18 months	155 (18.9)	0.25 (0.20 - 0.30)	0.43 (0.25 - 0.60)	0.17 (-0.01-0.35)
24 months	141 (19.1)	0.26 (0.20 - 0.31)	0.37 (0.21 - 0.53)	0.12 (-0.05-0.29)
30 months	131 (17.9)	0.23 (0.18 - 0.28)	0.38 (0.21 - 0.55)	0.15 (-0.03-0.32)
36 months	168 (21.9)	0.27 (0.22 - 0.32)	0.39 (0.22 - 0.56)	0.12 (-0.06-0.30)
42 months	108 (12.9)	0.16 (0.12 - 0.20)	0.20 (0.10 - 0.29)	0.04 (-0.07-0.14)
48 months	129 (15.8)	0.20 (0.16 - 0.25)	0.29 (0.15 - 0.42)	0.08 (-0.05-0.22)
54 months	89 (10.5)	0.15 (0.11 - 0.19)	0.10 (0.03 - 0.17)	-0.04 (-0.12-0.03)
60 months	80 (9.9)	0.14 (0.10 - 0.17)	0.12 (0.05 - 0.20)	-0.01 (-0.09-0.07)
66 months	67 (9.0)	0.11 (0.08 - 0.15)	0.12 (0.04 - 0.20)	0.01 (-0.07-0.09)
6 years	54 (6.8)	0.09 (0.06 - 0.12)	0.09 (0.03 - 0.16)	0.01 (-0.06-0.07)
6.5 years	44 (5.9)	0.07 (0.04 - 0.09)	0.11 (0.04 - 0.17)	0.04 (-0.03-0.11)
7 years	35 (4.3)	0.05 (0.03 - 0.08)	0.08 (0.02 - 0.14)	0.03 (-0.03-0.09)
8 years	37 (4.4)	0.05 (0.03 - 0.07)	0.08 (0.02 - 0.14)	0.03 (-0.03-0.09)

*Adjusted estimates come from a linear mixed effects model adjusted for clustering by child and for parental employment, household income, maternal education, household asset score, maternal BMI, maternal age, maternal depression, maternal intimate partner violence, maternal alcohol use, maternal smoking at cohort enrollment, birthweight zscore, birth length zscore, and site. Risk differences compare children exposed to HIV, but uninfected to children HIV unexposed.

Table S6. Overweight (body mass index-for-age-z-scores > 2) at individual time points overall and by HIV exposure status.

	**Overall** N=1070<br>Unadjusted n (%)	**Children HIV unexposed** n=235<br>Adjusted Prevalence 95% CI*	**Children HIV exposed uninfected** n=835<br>Adjusted Prevalence 95% CI*	Adjusted Prevalence Difference* (95% CI)
6 months	125 (13.8)	0.15 (0.12 - 0.18)	0.14 (0.08 - 0.20)	-0.01 (-0.07-0.06)
9 months	113 (13.9)	0.17 (0.13 - 0.20)	0.12 (0.07 - 0.17)	-0.04 (-0.11-0.02)
12 months	75 (9.8)	0.11 (0.09 - 0.14)	0.11 (0.06 - 0.16)	0.00 (-0.06-0.06)
18 months	188 (23.1)	0.25 (0.21 - 0.29)	0.24 (0.17 - 0.32)	0.00 (-0.09-0.09)
24 months	76 (10.3)	0.11 (0.08 - 0.14)	0.13 (0.07 - 0.18)	0.02 (-0.05-0.08)
30 months	105 (14.3)	0.17 (0.13 - 0.21)	0.16 (0.10 - 0.22)	-0.01 (-0.08-0.06)
36 months	69 (9.0)	0.09 (0.06 - 0.11)	0.11 (0.06 - 0.16)	0.02 (-0.04-0.08)
42 months	60 (7.2)	0.07 (0.05 - 0.10)	0.08 (0.04 - 0.12)	0.01 (-0.04-0.06)
48 months	43 (5.3)	0.05 (0.03 - 0.07)	0.07 (0.04 - 0.11)	0.02 (-0.02-0.07)
54 months	39 (4.6)	0.05 (0.03 - 0.07)	0.04 (0.01 - 0.07)	-0.01 (-0.04-0.02)
60 months	33 (4.2)	0.04 (0.03 - 0.06)	0.03 (0.00 - 0.05)	-0.01 (-0.04-0.02)
66 months	27 (3.7)	0.04 (0.02 - 0.06)	0.03 (0.00 - 0.06)	-0.01 (-0.04-0.02)
6 years	33 (4.2)	0.05 (0.03 - 0.07)	0.03 (0.00 - 0.05)	-0.03 (-0.06-0.01)
6.5 years	38 (5.1)	0.06 (0.04 - 0.08)	0.03 (0.00 - 0.06)	-0.03 (-0.07-0.00)
7 years	39 (4.9)	0.06 (0.04 - 0.08)	0.02 (-0.00 - 0.04)	-0.04 (-0.07--0.01)
8 years	43 (5.1)	0.07 (0.05 - 0.09)	0.02 (0.00 - 0.05)	-0.04 (-0.07--0.01)

*Adjusted estimates come from a linear mixed effects model adjusted for clustering by child and for parental employment, household income, maternal education, household asset score, maternal BMI, maternal age, maternal depression, maternal intimate partner violence, maternal alcohol use, maternal smoking at cohort enrollment, birthweight zscore, birth length zscore, and site. Risk differences compare children exposed to HIV, but uninfected to children HIV unexposed.

Figure S1. Directed Acyclic Graph.

Except for birth weight and length z score, all covariates measured during pregnancy. Multivariable models included parental employment, household income, maternal education, household asset score, maternal body mass index, maternal age, maternal depression, maternal intimate partner violence, maternal alcohol use, maternal smoking, site, birth weight and length z-scores.

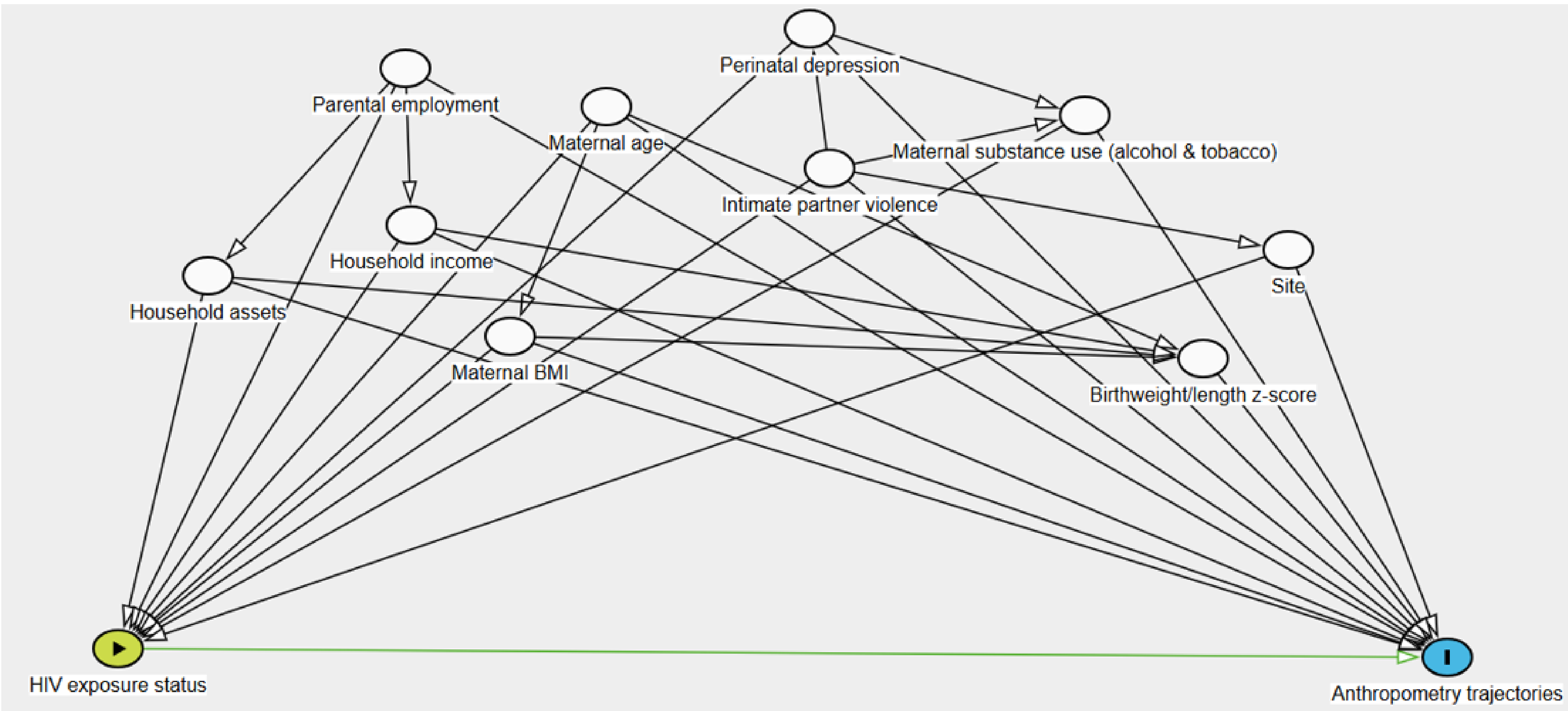

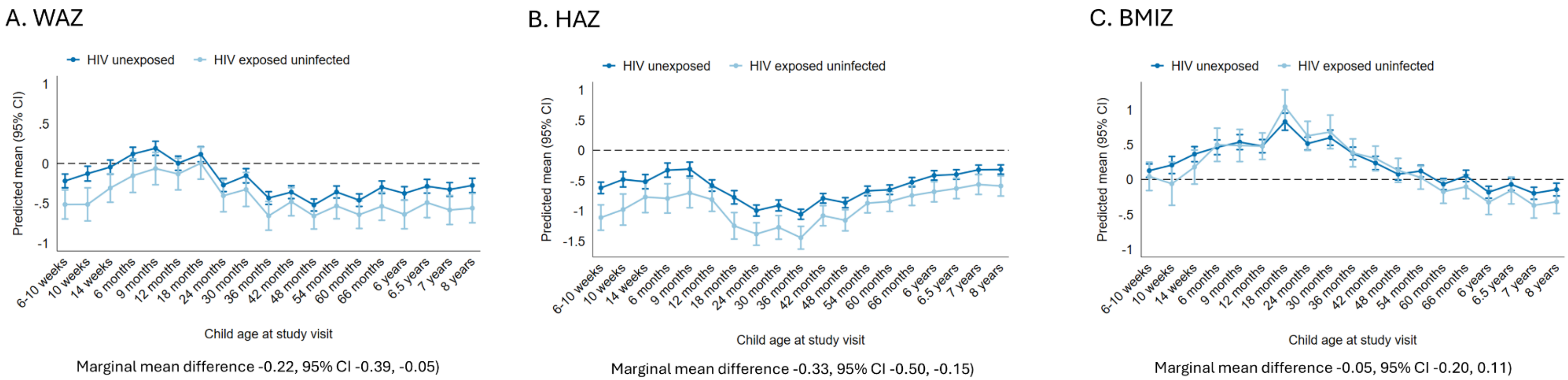

Figure S2. Weight for Age (WAZ), Height for Age (HAZ) and Body Mass Index (BMIZ) scores by HIV exposure status from 6 weeks to 8 years of age, excluding covariates measured at birth. Means (95% CI) at individual time points and marginal (overall) mean differences (95% CI) from linear mixed effects models adjusted for clustering by child and for parental employment, household income, maternal education, household asset score, maternal body mass index, maternal age, maternal depression, maternal intimate partner violence, maternal alcohol use, maternal smoking at cohort enrollment, and site.

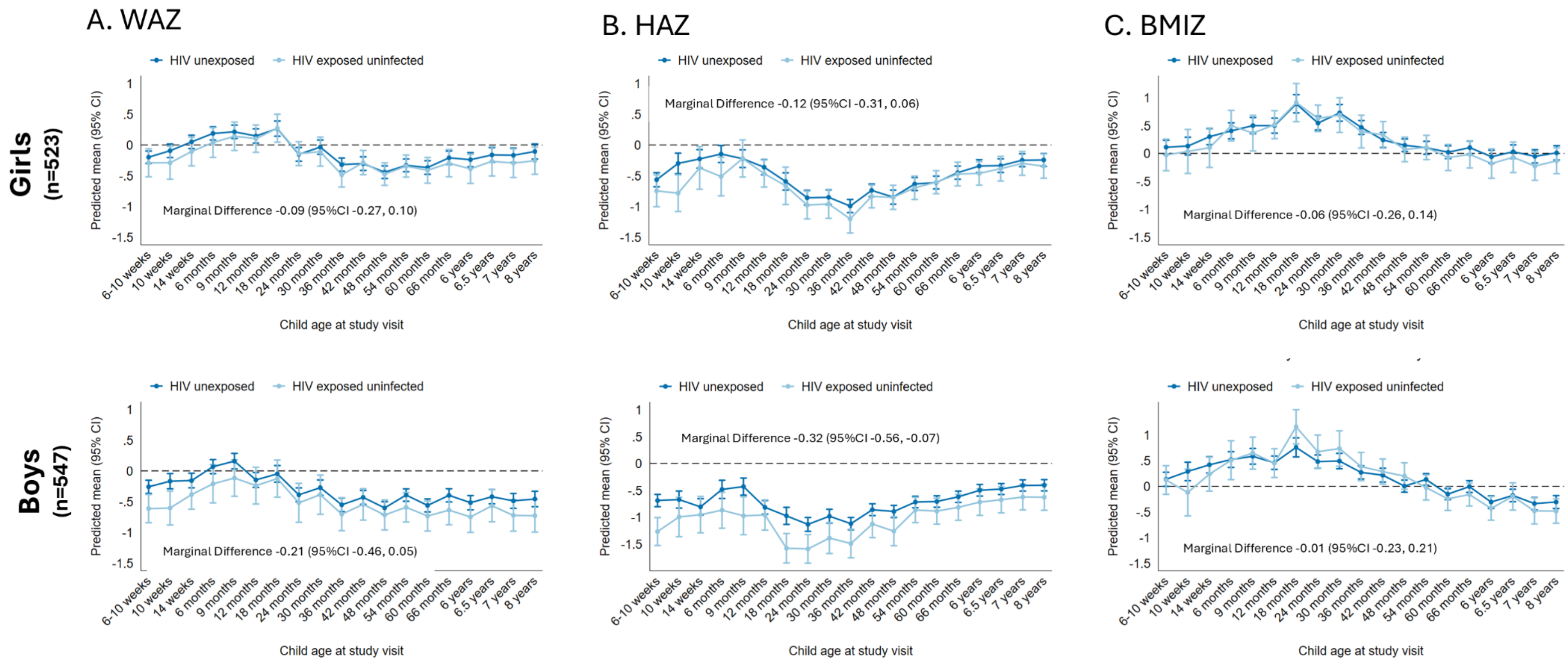

Figure S3. Weight for Age (WAZ), Height for Age (HAZ) and Body Mass Index (BMIZ) scores stratified by infant sex.

Multivariable linear mixed effects models are adjusted for parental employment, household income, maternal education, household asset score, maternal body mass index, maternal age, maternal depression, maternal intimate partner violence, maternal alcohol use, maternal smoking at cohort enrollment, birthweight zscore, birth length zscore, and site.

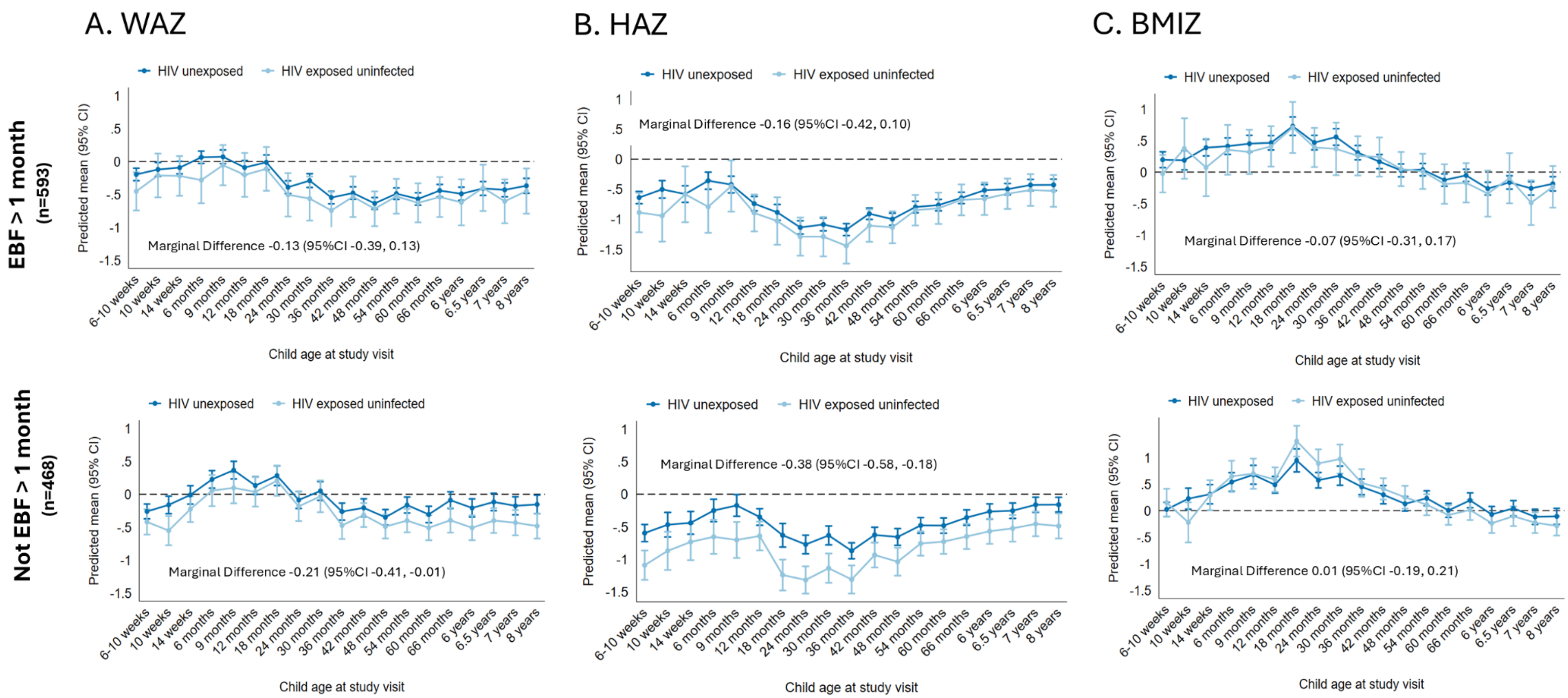

Figure S4. Weight for Age (WAZ), Height for Age (HAZ) and Body Mass Index (BMIZ) scores stratified by exclusive breastfeeding status (EBF). Multivariable linear mixed effects models are adjusted for parental employment, household income, maternal education, household asset score, maternal body mass index, maternal age, maternal depression, maternal intimate partner violence, maternal alcohol use, maternal smoking at cohort enrollment, birthweight zscore, birth length zscore, and site.

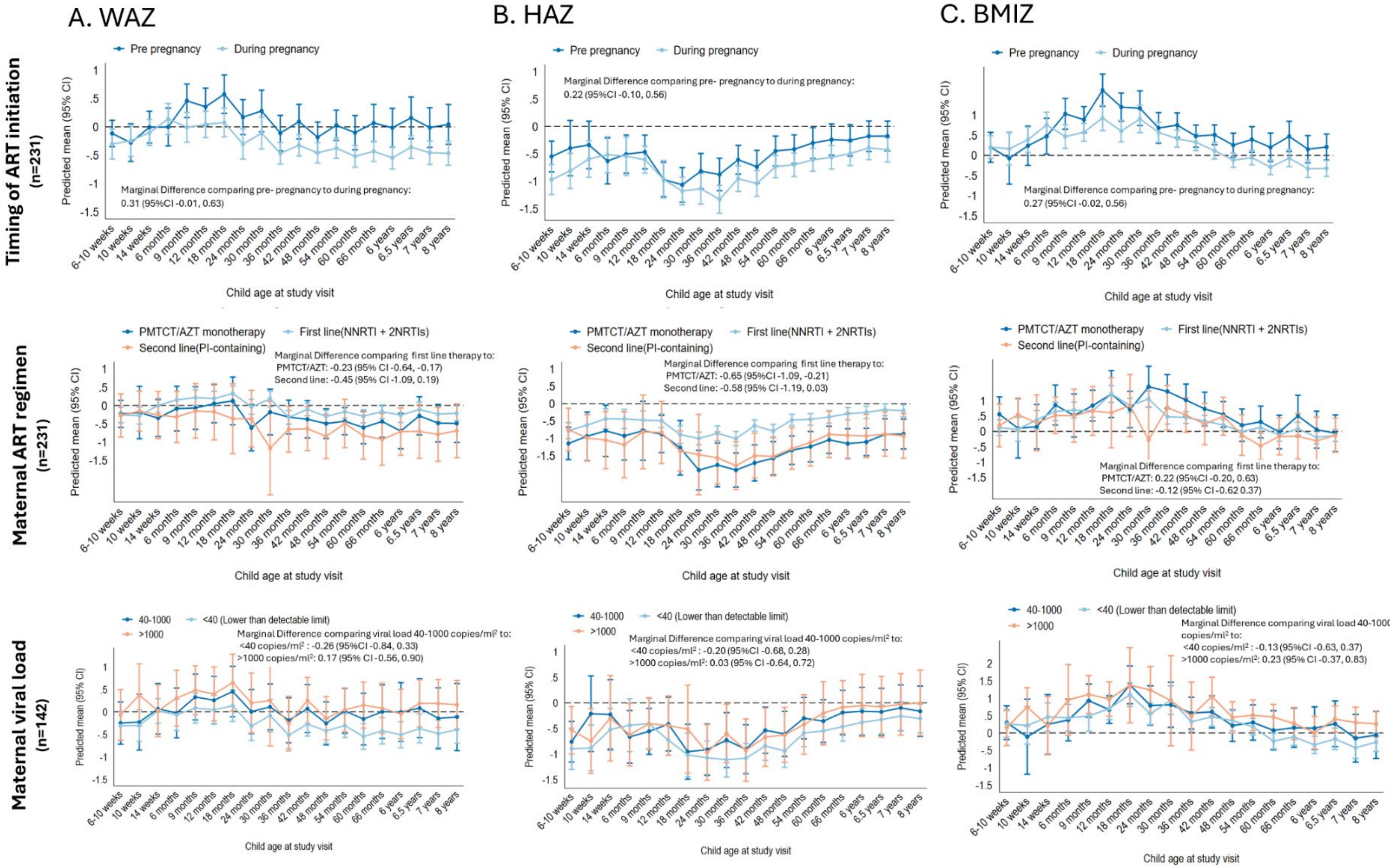

Figure S5. Subgroup analyses of Weight for Age (WAZ), Height for Age (HAZ) and Body Mass Index (BMIZ) scores among HIV exposed, but uninfected children. Multivariable linear mixed effects models are adjusted for parental employment, household income, maternal education, household asset score, maternal body mass index, maternal age, maternal depression, maternal intimate partner violence, maternal alcohol use, maternal smoking at cohort enrollment, birthweight zscore, birth length zscore, site, and CD4 count during pregnancy.

Figure S6. Sankey plot of change in stunting (HAZ < -2SD) and overweight (BMIZ > 2 SD) status by age and HIV exposure status. Numbers on the graph represent the proportion moving from one category to another category, defined by change in overweight or stunting status, from their last study visit. For this analysis, stunting is defined starting at 12 months of age, therefore change in stunting status is available starting at 18 months of age. Overweight is defined starting at 6 months of age, therefore, change in overweight status is defined starting at 9 months of age. *Sample interpretation*: From 6 to 9 months of age, 78% of infants who are normal weight stay normal weight, 8% move from normal to overweight, another 8% move from overweight to normal weight, and 6% remain overweight. Graphs are presented overall and by HIV exposure status for change in stunting status (A-C) and change in overweight status (D-F). HU: children HIV unexposed; HEU: children exposed to HIV, but uninfected.

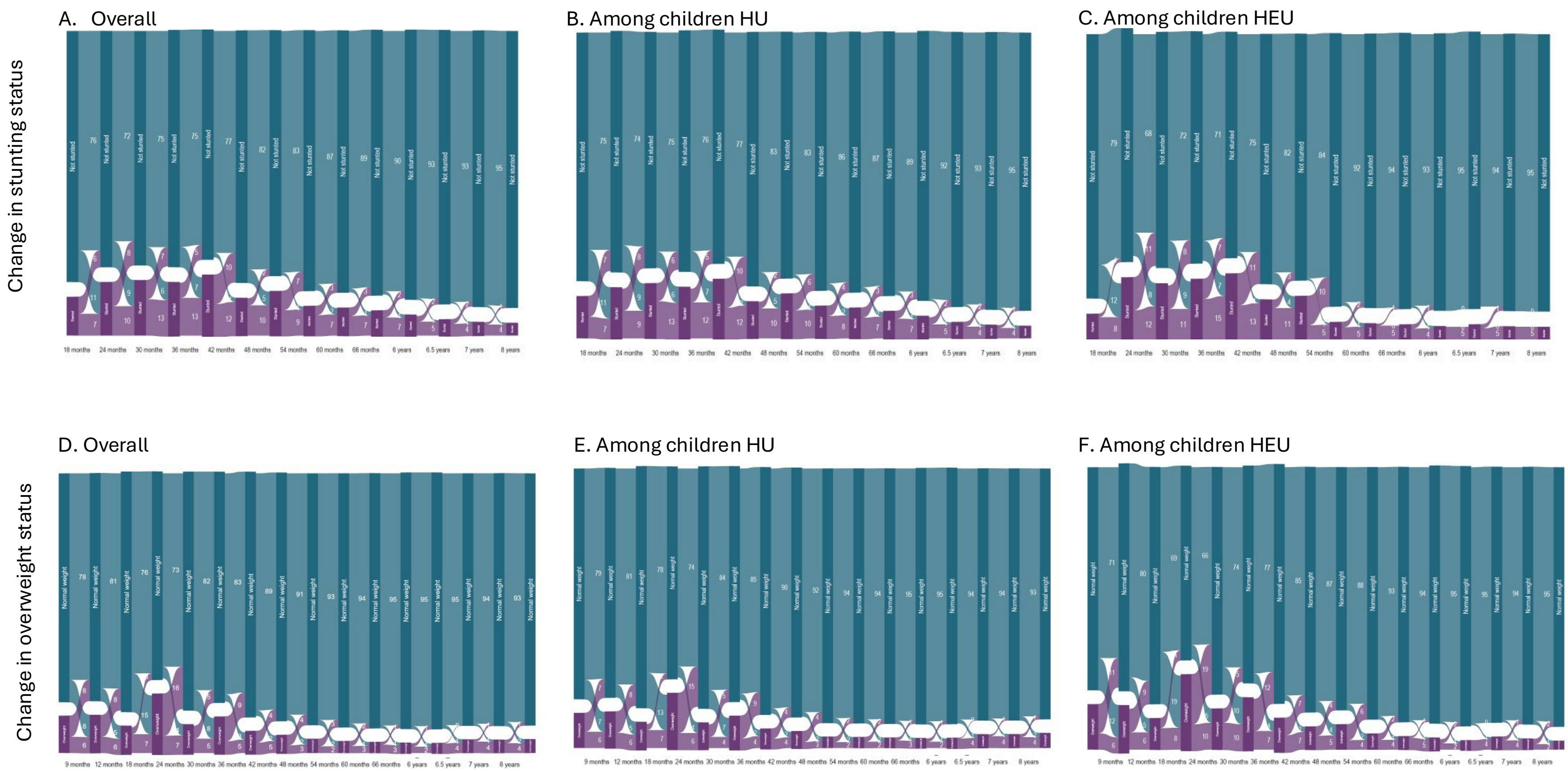

References

1. City of Cape Town. HIV and TB Plan 2013/2014. 2014.
2. Pellowski J, Wedderburn C, Stadler JAM, Barnett W, Stein D, Myer L, et al. Implementation of prevention of mother-to-child transmission (PMTCT) in South Africa: outcomes from a population-based birth cohort study in Paarl, Western Cape. BMJ Open. 2019;9(12):e033259.
3. Zar HJ, Barnett W, Myer L, Stein DJ, Nicol MP. Investigating the early-life determinants of illness in Africa: the Drakenstein Child Health Study. Thorax. 2015;70(6):592-4.
4. Beauclair R, Petro G, Myer L. The association between timing of initiation of antenatal care and stillbirths: a retrospective cohort study of pregnant women in Cape Town, South Africa. BMC Pregnancy Childbirth. 2014;14:204.
5. Melamed N, Hiersch L. Fetal Growth in Twin Pregnancies and the Choice of Growth Chart. Matern Fetal Med. 2022;4(4):234-7.
6. Wilson RS. Twin growth: initial deficit, recovery, and trends in concordance from birth to nine years. Ann Hum Biol. 1979;6(3):205-20.
7. Womack SR, Beam CR, Giangrande EJ, Scharf RJ, Tong X, Ponnapalli M, et al. Nonlinear Catch-Up Growth in Height, Weight, and Head Circumference from Birth to Adolescence: A Longitudinal Twin Study. Behav Genet. 2023;53(5-6):385-403.
8. Zar HJ, Pellowski JA, Cohen S, Barnett W, Vanker A, Koen N, et al. Maternal health and birth outcomes in a South African birth cohort study. PloS one. 2019;14(11):e0222399-e.
9. Rickman RR, Widen EM, Lane CE, Abrego MR, Nichols AR, Foster SF, et al. Infant body composition trajectories differ by in utero exposure to gestational diabetes mellitus: a prospective cohort from birth to 12 months. Am J Clin Nutr. 2025;121(1):40-9.
10. (UNICEF) WHOatUNCsF. Recommendations for data collection, analysis and reporting on anthropometric indicators in children under 5 years old. Geneva: WHO and UNICEF; 2019.
