# Supplementary material for: In-utero HIV exposure and postnatal growth to 8 years of age: a prospective cohort study in the Western Cape, South Africa
Source: Lancet Child Adolesc Health. 2026 Sep;10(9):658–67. doi: 10.1016/S2352-4642(26)00121-5 (PMC13437777; doi:10.1016/S2352-4642(26)00121-5)
Supplement: Supplementary file 2 [file mmc2.pdf]

THE LANCET
Child & Adolescent Health

Supplementary appendix 2

This Equitable Partnership Declaration (EPD) was submitted by the authors, and we reproduce it as supplied. It has not been peer reviewed. *The Lancet*'s editorial processes have not been applied to the EPD.

Supplement to: Bengtson AM, Pellowski JA, Botha M, et al. In-utero HIV exposure and postnatal growth to 8 years of age: a prospective cohort study in the Western Cape, South Africa. *Lancet Child Adolesc Health* 2026; published online July 17. https://doi.org/10.1016/S2352-4642(26)00121-5.

<u>Equitable Partnership Declaration</u>

If any questions do not apply to your study, please indicate "N/A" for "not applicable.
For more information on how to complete this form see the Information for Authors document.

<u>Researcher considerations</u>

1. Please detail the involvement that researchers who are based in the country or countries of study had during a) study design; b) clinical study processes, such as processing blood samples, prescribing medication, or patient recruitment; c) data interpretation; and d) manuscript preparation, commenting on all aspects. If they were not involved in any of these aspects, please explain why.

   This should include a thorough description of their leadership roles in the study. Are local researchers named in the author list or the acknowledgements, or are they not mentioned at all (and, if not, why)? Please also describe the involvement of early career researchers based in the location of the study. Some of this information might be repeated from the Contributors section in the manuscript. Note: we adhere to ICMJE authorship criteria for naming authors on a paper.

**a) Study design:**<br>HJZ conceptualized and is the overall PI of the Drakenstein Child Health Study (DCHS), with DJS conceptualizing and leading elements related to psychosocial health. Both are South Africans. HJZ and EG (also South African) helped to design the present analysis, with input from other co-authors. Six of 10 authors are South African, representing the study leadership and operational teams.
**b) Clinical study processes:**<br>All data clinical and field work activities were conducted by investigators based in South Africa. MB (South African) was the medical officer overseeing clinical aspects of the study and TB (who lives in South Africa) was the project manager overseeing study activities. As mentioned, HJZ is overall PI of the DCHS. AMB and JP (both USA) worked with LW and HJZ (both South African) on the specific grant supporting analyses reported here.
**c) Data interpretation:**<br>Data cleaning was led by LW (South African) with input from the DCHS data staff, including clinical input from MB, EG, and HJZ. Data analysis and interpretation were done by AMB (USA). All authors assisted with interpretation of the study findings and were given the opportunity to comment on these interpretations.
**d) Manuscript preparation:**<br>The manuscript was drafted by AMB (USA) with input from HJZ, LW, TB, MB, EG (all South African), as well as JP (USA), and DB and TM (Australia).

2. How was funding used to remunerate and enhance the skills of researchers in the countries of study? And how was funding used to improve research infrastructure at the study sites?

Potentially effective investments into long-term skills and opportunities within local institutions could include training or mentorship in analytical techniques and manuscript writing, opportunities to lead all or specific aspects of the study, financial remuneration rather than requiring volunteers, and other professional development and educational opportunities.

Improvements to research infrastructure could include funding extended trial designs (eg, platform trials), establishment of long-term contracts for research staff, building research facilities, and setting up local control of funding allocation.

**Skills:** Funding was used to upskill local clinical staff and staff in the data and administrative teams in aspects of clinical measures and data acquisition including anthropometry measurements and in analyses.
**Research infrastructure:** Funding for this study supported the procurement of equipment to measure indicators of cardiovascular health (i.e. arterial stiffness and pulse wave velocity) in children at both study sites, enhancing the ability to evaluate cardiometabolic health in paediatric populations within study communities. Most research staff involved in the study are on full time permanent contracts.

3. How did you safeguard the researchers who implemented the study?

Please describe how you guaranteed safe working conditions for study staff, including provision of appropriate personal protective equipment, protection from violence, and prevention of overworking.

Personal protective equipment (PPE) was always available for clinical activities. Contracts for research staff limited their working time to 35-hour working weeks Monday to Friday. Staff work as part of well established research teams and collaborate closely with staff in the Western Cape Health Department clinical services. Community workers from the same communities form part of the research team and regularly advise on issues of safety.

<u>Benefits to the communities and regions of study</u>

4. How does the study address the research and policy priorities of its location?

How were the local priorities determined and then used to inform the research question? Who decided which priorities to take forward? Which elements of the study address those priorities?

This is a low socio-economic community with a high prevalence of adults living with HIV. The impact of maternal HIV on child health outcomes in uninfected children is a key health issue and a local priority. The DCHS comprehensively addresses the determinants of child health

in a poor socio-economic community to inform area for strengthening health and preventing illness. Growth is a key indicator of child health and is also an area amenable to intervention, making it a priority in this research.

5. How will research products be shared in the community of study?

For instance, will you be providing written or oral layperson summaries for non-academic information sharing? Will study data be made available to institutions in the region(s) of study? The Lancet Child & Adolescent Health *encourages authors to translate the summary (abstract) into relevant languages after paper editing; do you intend to translate your summary?*

We will publicize and make the research paper available to all staff at each study site. Results will also be made available to the local health authorities and the Western Cape Health Dept as well as to a local health initiative that focuses on the first 1000 Days. In addition, feedback to participants and their families is periodically provided in the DCHS.

6. How were individuals, communities, and environments protected from harm?

a) *How did you ensure that sensitive patient data were handled safely and respectfully? Was there any potential for stigma or discrimination against participants arising from any of the procedures or outcomes of the study?*

All data were stored securely on cloud-based systems. Blood samples sent for analysis were accompanied by patient IDs but no other patient details. All data is anonymised and linked only through a study number HIV remains stigmatized within study communities; however DCHS did not conduct HIV testing (for HIV infection, CD4 count or viral load). Rather this information was garnered from the National Health Laboratory System in South Africa to minimize the potential for stigma.

b) *Might any of the tests be experienced as invasive or culturally insensitive?*

The venepuncture blood tests might be considered invasive, so we ensured these (and all other clinical processes and data collection measures) were accompanied by informed patient consent. However, we have been working in DCHS for approximately 15 years in this community with very high rates of acceptability and very high cohort retention and satisfaction with the study

c) *How did you determine that work was sensitive to traditions, restrictions, and considerations of all cultural and religious groups in the study population?*

Many of our research staff at site are from the local communities. We regularly engage with members of the local communities and with local schools to ensure that the work is done in a sensitive and culturally appropriate manner. Feedback from mothers in the study indicates very high levels of satisfaction with the study.

d) *Were biowaste and radioactive waste disposed of in accordance with local laws?*

Yes: biowaste was disposed of safely and appropriately. We did not generate radioactive waste.

e) *Were any structures built that would have impacted members of the community or the environment (such as handwashing facilities in a public space)? If so, how did you ensure that you had appropriate community buy-in?*

N/A: no structures were built

f) *How might the study have impacted existing health-care resources (such as staff workloads, use of equipment that is typically employed elsewhere, or reallocation of public funds)?*

Feedback on design the overall DCHS was sought from representatives of all sites included in the study, to ensure minimal disruption to clinical activities and to local workflows.
The study has reduced workload on the local health providers by optimising health of mothers and children and by careful surveillance for any ongoing issues. Therefore illness is generally detected early. In addition, there is a substantial amount of health education provided by the study to promote health and to avert disease.

7. Confirm that local ethics review was sought, and please provide the approval number. If not sought, please explain why.

We confirm that local ethics review was completed. The DCHS was approved by the Faculty of Health Sciences, Human Research Ethics Committee, University of Cape Town (401/2009) and by the Western Cape Provincial Health Research committee. HREC approval is renwed annually, and participants are re-consented annually

---

Secondary analyses

8. Have the data analysed in your study been extracted from another source, such as a national survey, rather than being directly collected by the authors of this paper?

N/A

If the authors of this paper were not involved in data collection, how were the findings interpreted with sufficient contextual knowledge?
The Lancet Child & Adolescent Health *believe contextual understanding is crucial for informed data analysis and interpretation.*

NA

---

9. Finally, please provide the title (eg, Dr/Prof, Mr/Mrs/Ms/Mx), name, and email address of an author who can be contacted about this statement.

**Name:** Angela Bengtson and Heather Zar<br>**Email:** angela.bengtson@emory.edu and heather.zar@uct.ac.za
